# Renal cancer associated with recurrent spontaneous pneumothorax in Birt-Hogg-Dubé syndrome: a case report and review of the literature

**DOI:** 10.1186/1752-1947-4-106

**Published:** 2010-04-19

**Authors:** Geoffrey Warwick, Louise Izatt, Elizabeth Sawicka

**Affiliations:** 1 Adult Intensive Care Unit, St Thomas' Hospital, London, UK; 2 Department of Clinical Genetics, Guy's Hospital, London, UK; 3 Department of Respiratory Medicine, Princess Royal University Hospital, Orpington, Kent, UK

## Abstract

**Introduction:**

Birt-Hogg-Dubé syndrome is a rare genodermatosis characterized by hair follicle hamartomas, renal tumors and spontaneous pneumothorax. We present the case of a patient with pulmonary cysts and recurrent spontaneous pneumothorax. She had typical skin lesions, and was found to have a hybrid oncocytoma which was surgically excised.

**Case presentation:**

A 60-year-old Caucasian woman had a 10-year history of cystic lung disease and recurrent spontaneous pneumothoraces. She was noted to have papular lesions over her face and forehead. The result of a biopsy showed these lesions to be fibrofolliculomas. A diagnosis of Birt-Hogg-Dubé syndrome was made and she was screened for renal tumors since these are a recognized association. A hybrid oncocytoma was detected which was surgically excised by partial nephrectomy.

**Conclusion:**

It is important to consider a possible diagnosis of Birt-Hogg-Dubé syndrome in cases of recurrent pneumothorax. Affected individuals must be screened for renal tumors, a potentially lethal consequence of this syndrome.

## Introduction

Birt-Hogg-Dubé syndrome (BHDS) is a rare genodermatosis characterized by hair follicle hamartomas, renal tumors and spontaneous pneumothorax. We present the case of a 60-year-old Caucasian woman with pulmonary cysts and recurrent spontaneous pneumothoraces who had typical skin lesions. On screening, she was found to have a hybrid oncocytoma which was surgically excised. A diagnosis of BHDS should be considered in cases of recurrent pneumothorax, and affected individuals must be screened for renal tumors.

## Case presentation

Our patient was a 60-year-old Caucasian woman who presented with recurrent left pneumothoraces. She also had mild scoliosis, an increased metacarpal index and mitral valve prolapse. There was a family history of mitral valve prolapse affecting both her mother and one of her daughters. Her brother has Usher's syndrome. Her parents were first cousins. An initial presumptive diagnosis of Ehlers-Danlos syndrome type IV was made but no confirmatory tests were carried out.

**Figure 1:**
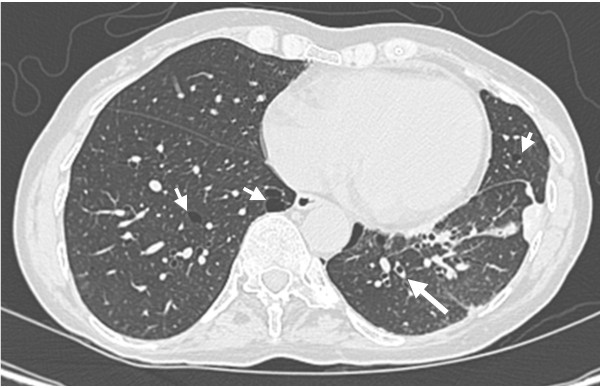
**Computed tomographic image of our patient's chest showing thin-walled cysts scattered throughout both the lower lobes in her lungs (small arrows) with some mild bronchiectasis (large arrow)**.

Over the next 10 years, our patient developed further pneumothoraces requiring video-assisted pleurodeses and bullectomy. Serial computed tomography (CT) scans showed the presence of cystic lung disease which was more marked at the bases; and development of mild bronchiectasis (Figure 1). In late 2004, she and her daughter, who was expecting her first child, were seen by clinical geneticists. On examination, it was found that our patient did not exhibit any of the expected complications of Ehlers-Danlos syndrome. The presumptive diagnosis was then reconsidered.

**Figure 2:**
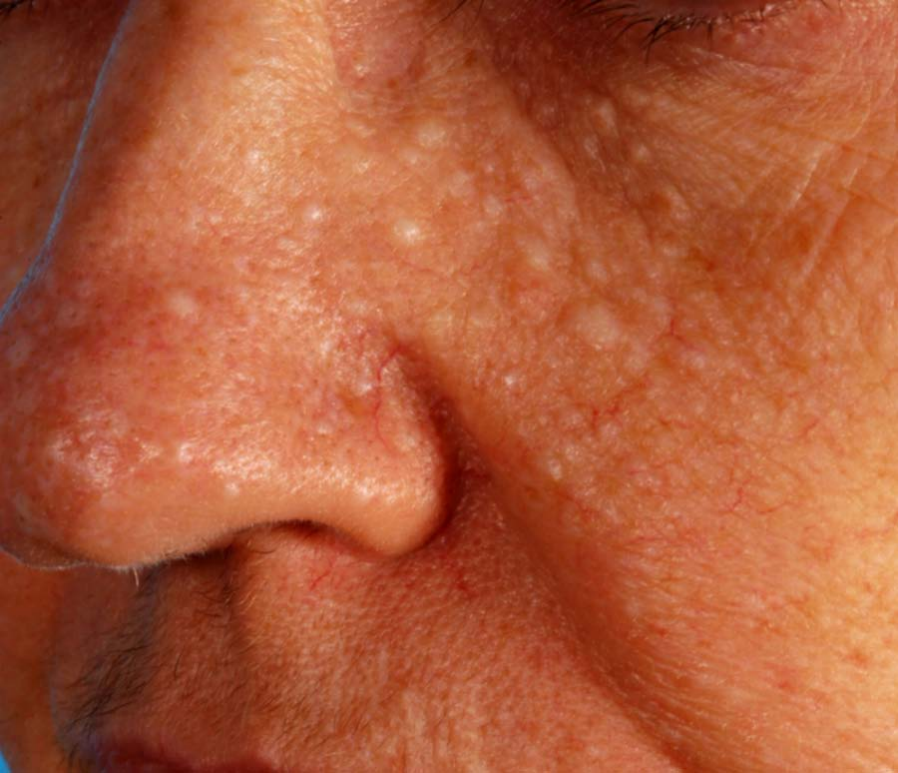
**Multiple pale, dome-shaped macules over our patient's face**. Histologically these were found to be fibrofolliculomas, the characteristic skin lesion of Birt-Hogg-Dubé syndrome.

**Figure 3:**
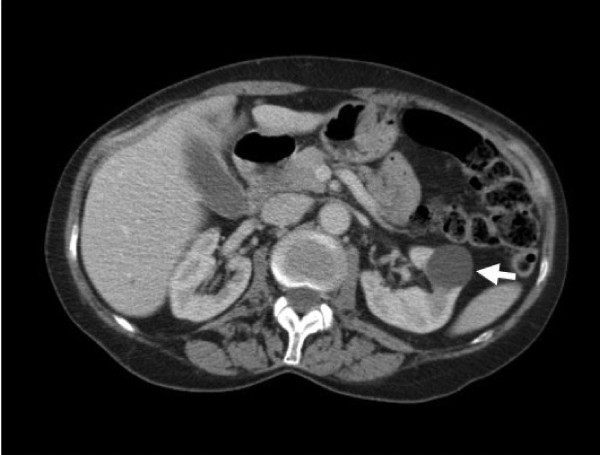
**Contrast-enhanced computed tomographic image of the abdomen showing a 1.7 cm non-enhancing lesion in the lower pole of the left kidney (arrow)**. This was excised by partial nephrectomy and found to be a hybrid oncocytoma, a tumor which is typical of Birt-Hogg-Dubé syndrome.

Our patient was noted to have pale, flat macules over her face and forehead (Figure 2), which had been present for at least 10 years. In conjunction with the recurrent pneumothoraces, this was suggestive of BHDS. A biopsy of a lesion from her neck confirmed the presence of a fibrofolliculoma, a characteristic skin finding. An abdominal CT scan (Figure 3) revealed a 1.7 cm lesion in the lower pole of the left kidney (T1 N0 M0). This was excised in a partial nephrectomy and was found to be a hybrid oncocytoma. The tumor was a 20 × 16 × 15 mm well-circumscribed, solid lesion with a macroscopically clear margin of 2 mm at the closest point. Microscopically, the nodule was predominantly composed of oncocytes interspersed with a smaller proportion of clear cells with uniform nuclei, and with focal cyst formation. The pathological stage was pT1a. Germline mutation analysis of the folliculin (FLCN) gene by polymerase chain reaction and sequencing identified a pathogenic mutation c.2052-2053 del, p.GlnfsX in exon 14. The mother of our patient and our patient's daughter carry the mutation and have fibrofolliculomas but no other phenotypic features. Our patient remains well and no further renal tumors have been detected.

## Discussion

In 1977, Drs Birt, Hogg and Dubé described 15 adults in a kindred of 70 who developed skin lesions which came on after the age of 25. They had multiple, small, dome-shaped papular skin lesions over the scalp, forehead, face and neck, and with scattered lesions observed on the chest and back [1]. Histologically, these were confirmed to be fibrofolliculomas and trichodiscomas, which are benign hamartomas of the hair follicle. Acrochordons (skin tags) were frequently associated as well, and this triad of skin lesions became known as BHDS. More recently, it has been suggested that these three lesions represent a spectrum of the same lesion, namely the fibrofolliculoma [2].

Subsequently, BHDS was found to be a marker for an internal disease. Case studies of recurrent pneumothorax, lung cysts [3] and renal tumors [3,4] have been reported before. A number of other phenotypic associations have also been described in case reports, in particular colonic tumors, but these have not been supported by the findings of larger case series [5].

Lung cysts are frequently seen in BHDS. A recent study found multiple pulmonary cysts in 89% of CT scans of BHDS patients [6]. Cysts in BHDS are typically discrete and well-circumscribed with normal intervening lung parenchyma. They are lined by a smooth, definable wall that does not enhance and are predominantly basilar and subpleural, though small intraparenchymal cysts can also be found [5]. As in our case, affected patients are at increased risk of spontaneous pneumothorax. The odds of this complication in BHDS patients are 50 times greater than in unaffected individuals [5]. In a series of 198 BHDS patients, 24% gave a history of pneumothorax, all of whom had lung cysts visible on chest CT imaging [6]. The risk of pneumothorax was statistically related to the number, the largest diameter and the largest volume of lung cysts. The association of bronchiectasis with BHDS, as in our case, has only been reported once before [7].

BHDS inheritance follows an autosomal dominant pattern [1]. It is caused by protein-truncating germline mutations of the FLCN gene (also known as BHD) which has been mapped to chromosome 17p11.2 [8]. More than 50 such mutations have been described, and these are mainly frameshift or nonsense mutations [9]. FLCN codes for a novel protein, folliculin, which is widely expressed in the kidney, lung and skin, and which has the characteristics of a tumor suppressor gene [10]. FLCN mutations have been identified in sporadic renal tumors [10], while mutations of FLCN have been detected in patients with sporadic [11] and familial [12,13] spontaneous pneumothorax without other phenotypic features of BHDS. Together with the recently-described FLCN-interacting protein (FNIP1), FLCN may function in pathways signaling through the mammalian target of rapamycin (mTOR). Such involvement with mTOR signaling is a feature of several hamartoma syndromes, including tuberous sclerosis complex, with which BHDS shares phenotypical characteristics [14].

Renal tumors have been reported in as many as 34% of individuals with germline FLCN mutations [9]. They are frequently multiple and bilateral and present at a mean age of 50.7 years [15]. The most common histological subtypes are hybrid oncocytic (50%) and chromophobe (34%) renal cell carcinomas. Clear cell oncocytomas and papillary renal cell cancers are less frequently found [15]. Radiographic screening is recommended, however no strictly-defined guidelines have been published. A typical strategy would involve abdominal CT and/or renal ultrasound at the time of diagnosis followed by interval screening every three to five years [16]. Nephron-sparing surgery is advocated, given the risk of further tumors developing [15].

## Conclusion

In our case, the recognition of characteristic skin lesions in the context of recurrent pneumothoraces and pulmonary cysts led to the diagnosis of BHDS. Subsequent CT screening identified a renal tumor which was then excised. It is important that BHDS should be considered in patients with recurrent spontaneous pneumothorax, particularly if skin lesions are present, as screening is essential to identify renal tumors. Families of index BHDS cases and patients with a family history of recurrent spontaneous pneumothorax should be considered for screening for the FLCN gene, even in the absence of other features, in view of the potentially lethal consequences.

## Competing interests

The authors declare that they have no competing interests.

## Authors' contributions

LI and ES were involved in the clinical management of our patient. GW performed the literature review and prepared the manuscript. LI and ES critically appraised the manuscript. All authors read and approved the final manuscript.

## Consent

Written informed consent was obtained from the patient for publication of this case report and any accompanying images. A copy of the written consent is available for review by the Editor-in-Chief of this journal.
